# Editorial: An integrative proposal in addiction and health behaviors psychosocial research: overview of new trends and future orientations

**DOI:** 10.3389/fpsyg.2025.1565681

**Published:** 2025-02-12

**Authors:** Álvaro García del Castillo-López

**Affiliations:** ^1^Analysis and Psychological Intervention in the Prevention of Health Risk Behaviours (PREVENGO) Research Group, Elche, Spain; ^2^Institute for Research on Drug Dependency (INID), Elche, Spain; ^3^Department of Health Psychology, Miguel Hernández University, Elche, Spain

**Keywords:** addictions, health behaviors, psychosocial research, alcohol, smartphone abuse, KAT, gambling, public health

Addiction and health behavior research is a multifaceted field that integrates perspectives from psychology, public health, neuroscience, and the social sciences. This Research Topic aims to present a comprehensive overview of current trends and future directions by highlighting critical factors that influence addiction and its prevention, treatment, and management. We received multiple submissions addressing various aspects of addiction and health behaviors, resulting in the publication of 10 articles.

Wan et al. developed a decision tree model to predict online shopping addiction and identified key contributing factors, including academic procrastination, social anxiety, and a diminished sense of purpose in life. Their study provides valuable insights for the development of targeted interventions aimed at mitigating online shopping addiction. Their results indicate that academic procrastination—rooted in avoidance behaviors and ineffective time management—emerges as a predominant factor predisposing individuals to compulsive online shopping. Furthermore, social anxiety, characterized by fear of judgment and avoidance of face-to-face interactions, serves as a significant contributor, with individuals resorting to online shopping as a coping mechanism. In addition, a diminished sense of purpose in life was found to exacerbate compulsive shopping behavior, as individuals seek fulfillment and distraction through online purchases. These findings point to the need for targeted intervention strategies that address the key psychological factors and behavioral patterns that drive online shopping addiction.

Using the Theory of Planned Behavior (TPB) as a conceptual framework, Atsbaha et al. examined the intention to chew khat among adolescents in Raya-Azebo District, Ethiopia. Khat chewing, a common social and cultural practice in Ethiopia, poses significant physical, psychological, and socioeconomic challenges. Despite various interventions to curb its use, the behavior remains widespread, especially among young people. This study aims to assess the factors that influence the intention to chew khat and provide evidence-based recommendations for prevention efforts. The results of the study show the importance of social influences and personal beliefs in shaping intentions to chew khat. To change positive attitudes toward khat chewing, the authors suggest strengthening social resistance skills and increasing self-efficacy. Educational programs, community engagement initiatives and policy implementation efforts are recommended to address the growing challenges associated with khat use in Ethiopia.

The relationship between cyberbullying and discrimination among students was explored in the work of Li Q. et al.. The authors examined the relationship between perceived discrimination and cyberbullying, identifying self-esteem as a mediator. Their findings suggest that students who experience discrimination are more likely to engage in cyberbullying behaviors, largely due to feelings of lowered self-esteem. In addition, the study identified self-compassion as a significant moderating factor that buffers the negative effects of perceived discrimination on self-esteem, thereby reducing the likelihood of engaging in cyberbullying. These findings suggest that interventions aimed at improving self-esteem and fostering self-compassion may be effective in reducing cyberbullying behavior. In addition, the study highlights the importance of creating inclusive and supportive environments within schools to reduce experiences of discrimination and promote psychological resilience among students.

Long et al. analyzed the influence of parental smartphone dependence on Internet addiction among elementary school students during the COVID-19 lock-in in China. Their study identified parent-child conflict as a mediating factor in this relationship, with parental roles moderating the observed effects. Specifically, fathers' dependence on smartphones was found to have a more significant influence on parent-child conflict than mothers' dependence. The results further suggest that parental smartphone overuse contributed to decreased parental involvement, weakened emotional bonds, and reduced overall family cohesion, all of which increased children's susceptibility to Internet addiction. In contrast, mothers, who were often the primary caregivers, were shown to mitigate some of these negative effects by providing emotional support and monitoring. These findings highlight the critical need for interventions aimed at reducing parental screen time, improving family communication, and promoting balanced use of technology in the home.

In an interesting focus on new modes of audiovisual consumption in social networks, Hu and Huang investigated the relationship between stress and short video addiction among Chinese users by extending the Compensatory Internet Use (CIU) model. The research introduces an extended CIU (E-CIU) framework that incorporates both compensatory motivations—such as the desire for social interaction and relaxing entertainment—and affective responses—like immersion and attitude—to better understand the mechanisms driving short video addiction. Key factors identified include emotional escapism, habit formation, and psychological dependence, with stress being the primary motivator that leads people to consume short videos as an emotional regulation mechanism. The findings suggest that the implementation of targeted interventions—such as digital detox initiatives and comprehensive stress management programs—can effectively reduce the risk of short video addiction and promote the adoption of healthier coping mechanisms.

Alcohol consumption remains a relevant global health problem. To analyze its incidence in the public sector, du Sartz de Vigneulles et al. conducted a qualitative study to explore the context of alcohol consumption among French public service employees, with the aim of understanding the underlying behavioral factors and identifying effective prevention measures. Using a qualitative research approach, the study examines the social, occupational, and personal factors that contribute to alcohol use disorders (AUD) in this specific occupational sector. The findings show that work-related stress, organizational culture and prevailing social norms play a significant role in shaping the drinking behavior of employees. In addition, the study identifies factors such as accessibility to alcohol and peer influence as critical contributors to workplace drinking. The authors emphasize the need for a comprehensive prevention strategy that includes organizational interventions such as educational initiatives, stress management programs, and policies that restrict the availability of alcohol in the workplace. These measures are essential to achieving a healthier and more productive workplace.

Li H. et al. examined the influence of family socioeconomic status (SES) on digital addiction in young children, identifying parenting style as a crucial mediating factor in this relationship. Their findings indicate that lower SES is associated with reduced parental involvement, increased exposure to digital devices, and a heightened risk of developing digital addiction. The study further highlights that authoritative parenting styles serve as a protective factor, effectively mitigating these risks, while permissive and neglectful parenting approaches exacerbate excessive screen time and problematic digital usage behaviors. Based on these insights, the authors advocate for socioeconomic interventions that prioritize parental education, improve access to resources, and promote healthy digital habits within families as key strategies to prevent digital addiction in children.

Another current major challenge among young people is the growth of online gambling. In this context, Suriá-Martínez et al. conducted a comprehensive analysis of the risk profile associated with online gambling among university students, with particular attention to the differences between disabled and non-disabled participants. Their findings indicate that students with disabilities are at increased risk of developing gambling-related problems, indicating an urgent need for specific preventive actions. The study identified several factors that contribute to online gambling behavior, including psychological distress, economic difficulties, and social influences. In particular, students with disabilities face additional challenges, such as social isolation and limited access to alternative recreational activities, which make them more vulnerable to problem gambling. Based on these data, the authors advocate for the implementation of tailored intervention programs at the university level, including financial literacy initiatives, mental health support services, and opportunities for social engagement. These findings underscore the importance of implementing proactive strategies to minimize gambling-related harm and support the development of healthier coping mechanisms among the student population.

Liu et al. investigated the impact of negative urgency on implicit mobile phone addiction among college freshmen, particularly in the context of social exclusion. Using a modified GO/NO-GO paradigm, the research examines how impulsivity driven by negative urgency influences students' susceptibility to compulsive mobile phone use and explores the moderating effects of social exclusion on this relationship. The findings of the study indicate that college freshmen with high levels of negative urgency exhibit a stronger tendency toward implicit mobile phone addiction compared to their low-negative urgency counterparts. Experiment 1 demonstrated a significant interaction between negative urgency levels and phone-related stimuli, revealing that individuals with high impulsivity struggle to inhibit responses to mobile phone-related content. Experiment 2 further established that social exclusion exacerbates this tendency, as students experiencing social isolation had even greater difficulty in controlling their engagement with phone-related stimuli. A three-way interaction effect was observed, highlighting the combined influence of negative urgency, social exclusion, and stimulus type in shaping mobile phone addiction tendencies. These findings underline the deterministic role of personality traits and environmental factors in the development of behavioral addictions among college students. By providing valuable insights into the interplay between psychological traits and social factors, this study contributes to the broader discourse on mobile phone addiction and highlights the need for holistic prevention strategies that address the unique challenges faced by college freshmen.

In this Research Topic, we have collected some of the most relevant trends in the current context of health and addiction research. Based on the included studies, the need for interdisciplinary collaboration in the identification of health problems, the design of prevention strategies, and the development of intervention programs and policies is evident. As addictive behaviors continue to evolve in response to societal changes, future research should prioritize the development of integrative frameworks that address the psychological, social, and biological determinants of addiction. In addition, advances in artificial intelligence and big data analytics offer promising opportunities to delve deeper into patterns of addiction, enabling the development of predictive models and personalized treatment strategies.

